# Underestimated Risk Perception Characteristics of Drivers Based on Extended Theory of Planned Behavior

**DOI:** 10.3390/ijerph19052744

**Published:** 2022-02-26

**Authors:** Yunteng Chen, Xianyong Liu, Jinliang Xu, Huan Liu

**Affiliations:** 1 Shaoxing Communications Investment Group Co., Ltd., Shaoxing 312000, China; yun2022@hotmail.com; 2 School of Highway, Chang’an University, Xi’an 710054, China; 2020021050@chd.edu.cn

**Keywords:** traffic safety, risk perception characteristics, structural equation modeling, underestimated driving risk, theory of planned behavior, normlessness

## Abstract

Aggressive driving behaviors due to drivers’ underestimation of risks are one of the major causes of traffic accidents. Due to the complexity of factors influencing risk perception, the mechanism of risk underestimation remains unclear. In this study, the theory of planned behavior (TPB) was extended by adding a new variable, namely drivers’ normlessness, forming an extended TPB (ETPB) framework to analyze the factors influencing risk underestimation and the extent of their influence. A total of 376 drivers’ perceived characteristics of risk underestimation were collected through an online survey, and a structural equation model was applied to investigate the effects of normlessness, behavioral attitudes, subjective norm, and perceived behavioral control on the tendency to underestimate the risk. The results showed that the ETPB model can explain the variance in the underestimation risk behavior by 69%; perceptual behavior control, attitude, and subjective norm (in descending order) had significant positive effects on driver’s tendency to underestimate risk; the normlessness variable can directly promote attitude and underestimated risk behavior; drivers with low annual mileage, complete insurance coverage, and no prior accident experience were more likely to underestimate driving risk. The study contributes to understanding of risk perception characteristics and provide theoretical basis for reducing underestimated risk behavior.

## 1. Introduction

Domestic and international studies have shown that more than 90% of traffic accidents are due to human factors [1], and one of the reasons for this phenomenon is that drivers are generally prone to overestimate their driving ability and misjudge the risks in a road environment [2,3]. The difference between a driver’s perceived subjective risk and objective risk influences their choice of driving behavior [4]. Drivers may engage in aggressive driving behavior or fail to avoid hazards in a timely manner if their subjective level of perceived risk is low. Related studies have pointed out that drivers who accurately judge risks and react appropriately can significantly reduce the occurrence of traffic accidents, whereas drivers who underestimate driving risks tend to be more prone to unconscious unsafe behaviors. Hence, it is important to study the factors influencing a driver’s underestimation of the risk to improve road safety.

Since the development of research in traffic safety, the factors influencing a driver’s tendency to underestimate the risk have been of interest to researchers in various countries, and some generally accepted, well-documented results and conclusions have been obtained. These studies mainly focused on the influence of driver characteristics on driver risk assessment. The differences between individual driver characteristics are found to affect their risk assessment, with driver gender being one of the main variables related to drivers, and its effect on risk assessment has been validated in many studies. Male drivers typically underestimate the potential risks in a traffic environment and are more likely to engage in unsafe driving behaviors [5,6,7]. In fact, behaviorally relevant studies have found that the high interest in cars and driving traditionally exhibited by males may lead to higher levels of skill and knowledge [8], which may lead to a high degree of confidence in their driving ability, whereas female drivers are more cautious and therefore make fewer violations than male drivers; however, female drivers are prone to make more operational errors while driving [9]. Overconfidence can reduce a driver’s risk perception and adversely affect driving performance [10,11]. Age differences have also been found in the tendency to underestimate risk; Rundmo and Iversen found that young drivers are slower and less efficient at detecting hazards [12]. This may be due to their driving inexperience and their weak perception of risk, which increases their tendency to underestimate the risk [13]. Compared to younger drivers, older drivers tend to overestimate the risk and have difficulty detecting unexpected, complex hazards. The risk-adaptation theory (RAT), however, states that a driver’s perception of risk is negatively related to his/her experience. The theory suggests that experienced drivers tend to have higher levels of risk acceptability, making it easier for them to underestimate the magnitude of objective risks [14]. There are significant differences in the attitudes toward risk and risk classification guidelines among experienced drivers. Because of these differences, novice drivers are often overwhelmed when faced with risks, whereas skilled drivers can quickly identify risks and take effective measures in a timely manner. There is an evident cross-talk between single factors, such as gender, age, and experience, which lack scientific validity as independent factors for judging a driver’s underestimation of risk. Meanwhile, objective factors, such as the gender, age, and experience, affect a driver’s psychological intrinsic factors to varying degrees [15,16], in turn affecting the estimation level of the driving risk. However, few studies have explained risk perception mechanisms in terms of the intrinsic factors. Hence, it is necessary to analyze the mechanisms of a driver’s tendency to underestimate the risk in terms of the intrinsic factors such as psychology and personality.

Previous studies have shown that the tendency to underestimate risk has multifactorial properties and is inseparable from the intrinsic characteristics of drivers. The theory of planned behavior (TPB) has emerged as a solution to address this issue; it is based on the core idea that behavioral intention is a determinant of behavior and that three cognitive factors, namely the attitude, subjective norm, and perceived behavioral control, jointly influence behavioral intention [17]; the stronger the behavioral intention, the more likely the manifestation of the behavior. Currently, the TPB has been widely used to explain traffic behaviors, such as fatigue driving [18], use of mobile communication devices while driving [19,20,21], yielding behavior [22], and lane grabbing [23]. The TPB has shown good validity in explaining various human behaviors [24,25]; for example, combined with the theory of reasoned action (TRA) and TPB studies in a meta-analysis, Sutton showed that these theoretical models explained, on average, 40–50% of the variance in intentions and 19–38% of the variance in behavior [24]. This finding is generally accepted in human behavior research [25]. In summary, the TPB seems to be a feasible approach to study a driver’s underestimation of risky behaviors by examining individual characteristics to analyze specific behaviors and thus improve the predictability of driver intentions [23]. It has been established that the stronger the driver’s perceived behavioral control, the more likely the driver is to underestimate driving risks [26]. In addition, there is a significant correlation between risky driving attitudes and risk perception [27]. Hence, the TPB model can provide a technical support to explore the psychological factors associated with the underestimation of risky driving behavior.

Based on the TPB, this study aimed to thoroughly investigate the tendency of drivers to underestimate driving risk, analyze the mechanism whereby the subjective perceived risk is lower than the objective risk during driving, and improve the prediction accuracy of the underestimated risk influence model by adding a personality trait variable, namely the normlessness. The structural equation model (SEM) is used to test the influence model and finally put forward a quantitative explanation for risk underestimation and to propose targeted measures that can reduce this phenomenon while driving.

The rest of this paper is organized as follows: Section 2 introduces the hypotheses of the extended TPB (ETPB) and proposed models and related variables. Section 3 presents the research methodology and the data collected, including the specific items of the questionnaire, the distribution of the participants, and data analysis. Section 4 includes the questionnaire reliability test, and the results of the validating factor analysis, SEM, model hypothesis test, and significance test of the demographic characteristics on the underestimation of the risk behavior. Section 5 presents the discussion, including theoretical and practical implications. Section 6 concludes the paper.

## 2. Theoretical Background and Model Hypothesis

**Figure 1:**
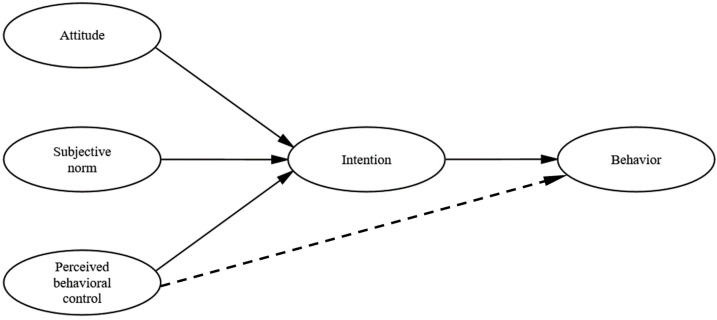
Model of the theory of planned behavior, dotted line indicates the impact of actual behavioral control.

The TPB was developed from the TRA, and its maturation was marked by the publication of Ajzen’s paper “Theory of Planned Behavior” in 1991 [28]. The theory provides an important analytical framework for understanding and predicting individual social behaviors, and its core idea is that behavioral intention (IN) is a determinant of behavior, while three cognitive factors, namely the attitude (ATT), subjective norm (SN), and perceived behavioral control (PBC), jointly influence behavioral intentions [17]. Figure 1 shows the interrelationships between these variables. The ATT is an individual’s positive or negative evaluation of the behavior; SN is the social pressure felt by the individual to adopt a particular behavior or not. PBC is an individual’s perception of the ease or difficulty of performing a particular behavior. According to the TPB and considering the context of this study, the ATT is the driver’s positive or negative evaluation of the underestimated driving risk; SN is the driver’s perceived social pressure to underestimate the driving risk; the PBC is the driver’s perceived ease of taking an underestimated driving risk, i.e., an assessment of his or her own driving skills and the external environment. The ATT, to a certain extent, reflects a driver’s intention to drive at risk. This in turn influences the driver’s risky driving behavior [29]. In this study, the more favorable a driver’s attitude toward underestimating the risky behavior, the more likely the driver is to exhibit this behavior; conversely, if a driver has a negative attitude, the less willing he/she is, subjectively, to exhibit this behavior. The SN can reflect the influence of significant others or groups on individual behavioral decisions. When significant others around them strongly advocate a behavior and if they occur frequently, then drivers tend to be inclined to exhibit that behavior. The PBC reflects the ease of drivers in accomplishing the underestimation of the risk behavior; if drivers are subjectively confident in their driving skills and the objective road environment meets the requirements (e.g., low traffic volume), drivers are likely to have the intention to underestimate the risk, in turn prompting them to underestimate the risk. Therefore, this study selects the TPB as the theoretical framework to develop a research model explaining a driver’s tendency to underestimate the driving risk. The following hypotheses are proposed:

**Hypothesis** **1** **(H1).** *Attitude to underestimate the driving risk positively influences the intention to underestimate the driving risk*.

**Hypothesis** **2** **(H2).** *Subjective norm positively influences the intention to underestimate the risk*.

**Hypothesis** **3** **(H3).** *Perceived behavioral control positively influences the intention to underestimate the risk*.

**Hypothesis** **4** **(H4).** *The intention to underestimate the risk positively influences a driver’s underestimated risk behavior*.

Although the TPB is proven to be highly applicable in practice, it has certain limitations, such as its static explanatory nature [30] and its focus on rational reasoning. To address these, Conner suggested extending the theoretical model [31]. Guided by this idea, Li introduced sensation seeking and risk perception to explain and predict the risky driving behavior of truck drivers based on the TPB [32]. McBride et al. used the TPB combined with psychosocial factors to explore the intention of young drivers to text while driving [19]. Conner et al. incorporated moral norms, anticipated regret, and past behavior into the TPB to explore factors influencing the speeding behavior [33]. The introduction of these variables further developed the TPB and enhanced its ability to explain and predict behavioral intentions and behaviors in specific contexts. Personality traits were found to be significantly associated with behavioral intention to drive and driver risk perception [34,35,36,37]. In addition, personality traits in drivers have been also found to be associated with an increased perception of stress that can affect behaviors while driving [38]. Hence, this study considered incorporating personality traits into the TPB to enhance its explanatory and predictive power.

Normlessness, originally defined as an individual’s belief that it is acceptable to do anything that they can get away with [39], now refers to an individual’s disrespect for and noncompliance with social norms. Drivers who scored high in normlessness were found to frequently violate traffic rules and were more likely to underestimate driving risks because such drivers do not care about traffic rules and are more likely to engage in aggressive driving behavior [40]. Ulleberg et al. studied the prediction of risky driving behavior in terms of the personality, attitude, and risk perception in 1932 young drivers in Norway and found that different personalities had different effects on risk perception [40]. Those who scored higher in normlessness perceived lower risk of traffic accidents, showed negative attitudes toward traffic safety, and would frequently engage in risky driving behaviors. Nordfjærn and Şimşekoğlu et al. investigated the different effects of personality traits, risk perception, and cultural differences on attitudes and driving behaviors on traffic safety among Turkish and Iranian drivers through a questionnaire [41]. The results showed that personality traits, particularly the normlessness, were the main predictors of attitudes and driving behavior in both samples. Notably, the normlessness was the strongest predictor of traffic attitudes and behaviors, and individuals with high levels of normlessness would violate social norms to achieve their personal goals, and therefore, they were also more likely to violate traffic rules. Normlessness has a direct effect on the behavior of road users and can also play an indirect role in driver behavior through other factors (attitudes). Therefore, based on previous studies, the following hypotheses are made:

**Hypothesis** **5** **(H5).** *Normlessness positively influences the attitude of underestimating the risk*.

**Hypothesis** **6** **(H6).** *Normlessness positively influences a driver’s behavior of underestimating the risk.*

**Figure 2:**
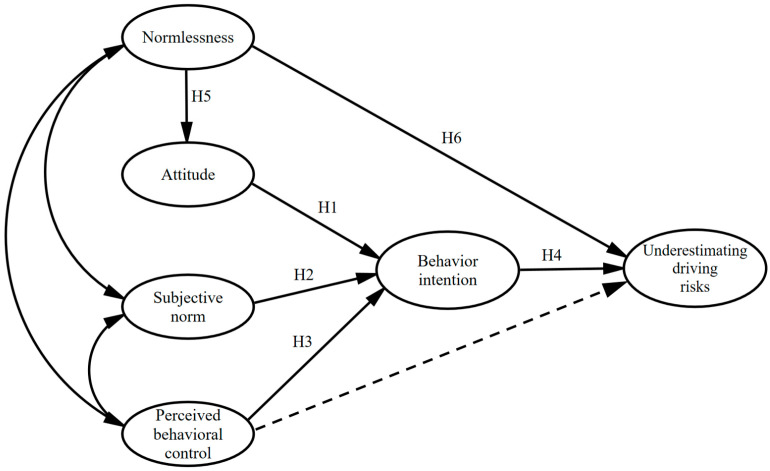
TPB model for driver underestimation of risk, dotted line indicates the impact of actual behavioral control.

Based on the above analysis, a research model combining the TPB and normlessness was proposed to explain the tendency of drivers in underestimating the driving risks. Figure 2 shows the research model with the above six hypotheses.

## 3. Materials and Methods

### 3.1. Questionnaire Design

Based on a review of the relevant literature, a multidisciplinary team composed of experts in the field of transportation, psychology, linguistics, and sociology conceived and drafted the first draft of the questionnaire. First, to ensure that the questionnaire is understood in a standardized manner, a driver’s underestimation of risk behavior is first explained. Driver underestimation of risk refers to a state of driver perception in which the driver believes that the risk arising from aggressive driving behavior is less than the objective risk [42]. For example, many drivers believe that traffic police may not be on duty at the location where he/she performs risky driving behaviors, that there are no electronic probes or photo violations nearby, and there is a tendency to overestimate their driving skills, believing that they can perform aggressive driving behaviors very easily without getting into trouble or endangering others. Then, the components of the questionnaire were identified by the team. The first part collected demographic characteristics of the participants, including gender, age, miles driven per year, education, insurance purchase status, and whether they had experienced an accident. The second part was a measure of the theoretical structure of the model, including the TPB scale, normlessness scale, and underestimated risk behavior scale.

The TPB scale was developed mainly based on the methodology of Ajzen for constructing the TPB questionnaire and combined with the characteristics of this study [43]. It comprises four main constructs: attitude toward underestimating risk, subjective norm, perceived behavioral control, and intention to underestimate risk. The measures of attitudes included instrumental and affective attitudes, which are directly measured by two items. The measures of the subjective norm included injunctive and descriptive norms, which were directly measured by two items. The measures of the perceived behavioral control included self-efficacy and control, which were directly measured by two items. The measure of intention to underestimate the risk was directly measured by three items.

The content of the items to measure the normlessness was proposed by Kohn and Schooler, ranging from strict adherence to rules to an evaluation of whether the rules should be followed [39]. In this study, it was adapted to incorporate the underestimation of risky driving characteristics, and four items were selected to measure a driver’s normlessness. Among them, N4 is a reverse scoring question; the higher the score, the more likely the driver obeys and respects the traffic rules. To make the entire scale measure scores represent the same meaning, the response data of N4 were reverse scored in the subsequent analysis.

The scale of the underestimated risk behaviors is mainly based on 10 major traffic violations (failure to yield, speeding, driving without a license, drunk driving, failure to maintain a safe distance from the vehicle in front, traveling against traffic, violating traffic signals, driving under the influence of alcohol, illegal overtaking, and illegal meeting) issued by the National Public Security Bureau and related research [40,44]. It is compiled by selecting four items to measure a driver’s underestimated risk behaviors.

The six constructs of a driver’s underestimation of risk perception characteristics were based on 23 questions, as shown in Table 1, with a five-point Likert scale, where one indicates “strongly disagree” and five indicates “strongly agree”.

### 3.2. Survey Implementation

In this study, data were collected through a web-based survey method. Web-based questionnaires are an effective tool for collecting willingness information at a low cost. The questionnaire was created and published on a popular survey platform in China, Questionnaire Star (https://www.wjx.cn/ accessed on 8 December 2021) [23]. Before the questionnaire was officially distributed, the scale was extensively solicited from expert teachers, and expert validity tests and modifications were made to identify ambiguities in the questionnaire and modify them in time to ensure that the measurement content could be understood in a standardized manner. The questionnaire link was set to be accessed only once per user. The target population of the questionnaire was mainly Chinese non-professional drivers, and did not consider the non-Chinese drivers and professional drivers. The official questionnaire was distributed from 17 August 2021 to 21 August 2021, and all the participants were informed that the survey was anonymous, that no personal privacy was collected, and that the data would be used for academic research only. If the question specifying the choice of “partially agree” did not have “partially agree” selected, then the questionnaire was invalid and was excluded. Finally, the data of driving age was zero, and driving ages older than this age were excluded. A total of 398 questionnaires were obtained, 31 invalid questionnaires were excluded, and 367 valid questionnaires were finally collected, with an efficiency rate of 92.21%. The research was reviewed and approved by the Research Ethics Committee of Chang’an University, Shaanxi, China (No.2021/12). The research content strictly follows the Declaration of Helsinki.

### 3.3. Statistical Analysis

First, the reliability and validity of the questionnaire were analyzed using SPSS 25.0 (International Business Machines Corporation, New York, NY, USA) to eliminate question items that did not meet the requirements; the reliability was tested by calculating the internal consistency reliability coefficient Cronbach’s α of the scale. The validity was tested using the Kaiser–Meyer–Olkin (KMO) test and Bartlett’s sphericality. Second, a Pearson bivariate correlation analysis using SPSS 25.0 was conducted to determine the correlation between the variables in the ETPB model [22].

In the third step, the influence model of the underestimation of the driving risk was tested using the SEM, which is a method for establishing, estimating, and testing causal relationships between variables [23]; it comprises a measurement model and a structural model. Compared to conventional methods, the SEM has controlled measurement error and allows a statistical evaluation of the theoretical models [46,47]. In addition, studies have shown that SEM can help build more accurate models for driving behavior analyses [48]. Therefore, in this study, the SEM was selected to construct and test the driver underestimation risk behavior model. The confirmatory factor analysis (CFA) is a part of the SEM analysis and is used to verify the validity and reliability of latent variable measurements in the proposed research model. The measurement model should be analyzed before constructing the structural model because the measurement model can correctly reflect the latent variables or influencing factors of the study [49]. In this study, the CFA of the measurement model was performed using AMOS 23.0 (International Business Machines Corporation, New York, America). The convergent validity refers to the extent to which multiple observed variables of the same latent variable are in agreement. In this study, the composite reliability (CR) and the average variance extracted (AVE) were selected for the model convergent validity test. Fornell and Larcker suggested that the CR of each latent variable should exceed the required value of 0.7, the AVE should be greater than the critical value of 0.5, and the standardized factor loadings of the observed variables should be greater than 0.7 to ensure the convergent validity of each latent variable measure [50]. The discriminant validity refers to the extent to which the latent variables are empirically distinct from each other, and each latent variable measure has acceptable discriminant validity when the square root of the AVE of each latent variable is greater than the correlation between this latent variable and the other latent variables in the model [51]. In this study, the initial model of the factors influencing driver underestimation risk was constructed using AMOS 23.0, and the data of the influencing variables were inputted to the initial model and fitted for calculation and testing. When applying SEM as a validation of the theoretical models, a certain degree of fitness should be ensured. Based on previous studies and SEM application studies conducted in the transportation field [19,23,32,48,52], the overall fitness analysis of the model in this study was represented by the standardized residuals (SRMR), comparative fit index (CFI), Tucker–Lewis index (TLI), and root-mean-squared error of the approximation (RMSEA); the normed fit index (NFI) and goodness-of-fit index (GFI) were used to represent the comparison between the hypothetical model and the independent theoretical model; the chi-squared freedom ratio ($\chi^{2}/df$) was used to represent the weighted analysis of the model freedom ratio. The model achieves a goodness of fit when it is less than 3, the SRMR and RMSEA are less than 0.08, TLI and CFI are greater than 0.9, and NFI and GFI are greater than 0.9 [52].

At the end, using a one-way ANOVA to explore the effect of demographic factors on a driver’s underestimation of the driving risk behavior. A one-way ANOVA can test whether different levels of a factor variable can cause a significant difference in the dependent variable. The demographic factors examined in this study were the gender, age, annual mileage, education level, insurance other than mandatory insurance, and accident experience.

## 4. Results

### 4.1. Demographic Analysis

The basic information of the questionnaire included demographic information and the frequency of a driver’s underestimation of risks, as shown in Table A1 of the Appendix A. Overall, the ratio of male to female drivers was 1.74:1, and the age of the participants ranged from 18 to 60 years and above, with 74.5% of the drivers aged between 18 and 50 years. According to the data released by the National Bureau of Statistics of China in 2020, the male-to-female ratio of Chinese motorists in 2020 was 2.08:1, with 71.79% of the drivers aged between 26 and 50 [53]. Thus, the sample structure of this study is representative of the typical population of Chinese drivers. Most of the drivers drove more than 20,000 km per year. Their education level covered high school to graduate groups; 62.40% of the drivers had other types of insurance besides the compulsory one; 59.70% of drivers had no accident experience; only 25.6% of the drivers never underestimated driving risks during driving; and 5.20% of the drivers always underestimated the risks.

### 4.2. Reliability and Validity Analyses

Table 2 presents the test results of reliability and validity analyses. From Table 2, the Cronbach’s α values of the N, ATT, SN, PBC, IN, and URB were 0.890, 0.885, 0.863, 0.873, 0.847, and 0.878, respectively, and the total correlation coefficients of the corrected items were greater than 0.7. The Cronbach’s α values were all greater than 0.8, indicating that the questionnaire had a high reliability [54]. Except for the normlessness item N4, the reliability coefficients of the remaining items were lower than the overall reliability coefficients, and the Cronbach’s α of item N4 after deletion was 0.902 greater than the overall reliability coefficient of 0.890 for the normlessness; therefore, the item N4 was deleted, after which the reliability of the scale met the requirements.

As listed in Table 2, the coefficient result of the KMO test was 0.954, and the significance of the sphericity test was less than 0.05, indicating that the questionnaire had good validity.

### 4.3. Correlation Analysis

Table 3 presents the results of correlation analysis. From Table 3, there was a significant correlation between N, ATT, SN, PBC, IN, and URB. Therefore, it is feasible to use the SEM to explore the interaction between the ETPB model and driver underestimation risk behavior.

### 4.4. Confirmatory Factor Analysis

Table 4 presents the results of convergent validity. According to Table 4, all the observed variables are significant, with standardized factor loadings between 0.7 and 0.95. The combined reliability values of N, ATT, SN, PBC, IN, and URB are above 0.8, and the mean variance-extracted AVE is above 0.6, thus satisfying the requirement of the convergent validity.

The results of discriminant validity are shown in Table 5, the square roots of the AVE of N, ATT, SN, PBC, IN, and URB are greater than the correlations between each latent variable and the other latent variables in the model, thus satisfying the requirement of discriminant validity.

### 4.5. Model Verification

**Figure 3:**
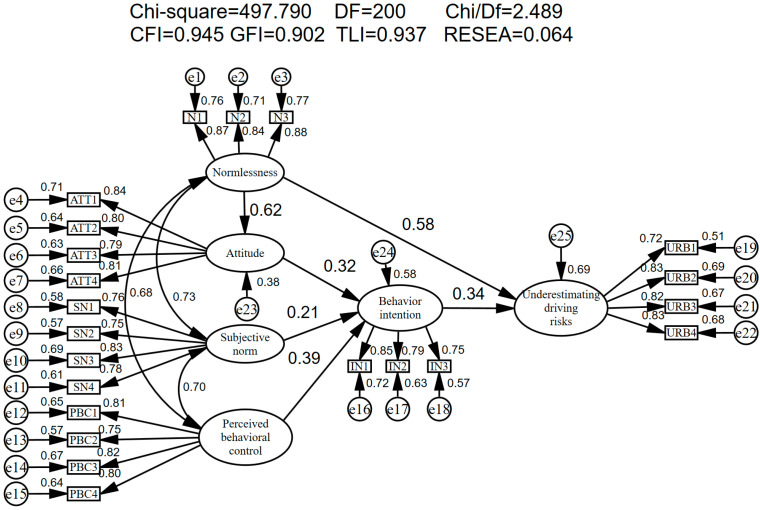
Modified model results.

Figure 3 shows the results of the modified model. As shown in Figure 3, the ATT, SN, and PBC can explain the variance in the intention to underestimate the risk by 58%, N can explain the variance in the ATT by 38%, and the model can ultimately explain the variance in the underestimated risk behavior by 69%, indicating that the research model has strong explanatory power for this behavior. Among them, the PBC and ATT have the most important effect on IN with standardized path coefficients of 0.39 and 0.32, respectively, and both are significant at the 1% significance level. This confirms that the PBC and ATT are the most important factors promoting a driver’s intention in underestimating the risk under the theoretical framework proposed in this study, followed by the SN with a standardized path coefficient of 0.21. This indicates that social pressure has a limited effect on a driver’s intention to underestimate the risk and that N has a significant effect not only on ATT but also on URB with standardized path coefficients of 0.62 and 0.58, respectively, indicating that a driver’s disrespect and disregard for traffic regulations affects the driver’s perception attitudes of the risk, prompting them to underestimate the objective risk of driving and predispose them to traffic accidents. The path coefficient of N on driver’s underestimation of the driving risk behavior is 0.58, which is greater than the path coefficient of intention on a driver’s underestimation of the risk behavior of 0.34, indicating that the newly added variable has the greatest influence on the driver’s underestimation of the risk behavior. This reveals that the less the driver pays attention to traffic regulations and fails to follow them, the more likely he/she is to underestimate the driving risk. Thus, the new variables added in this study have good explanatory power for driver’s intentions and behavior of underestimating the risk.

Table 6 presents the fitness index situation of the final model. All the indices conform to the model evaluation criteria; therefore, the model adequately fits the data.

The validity of the model hypotheses can be tested by the *p*-values of their path coefficients, the results of which are presented in Table 7, from which it can be seen that the standardized path coefficients of all the hypotheses are greater than 0 and meet the 0.01 level of significance, indicating that all the 6 hypotheses of the model are acceptable. Specifically, the underestimation risk attitude (β = 0.323, *p* < 0.001), subjective norm (β = 0.214, *p* = 0.006), and perceived behavior control (β = 0.387, *p* < 0.001) positively affect a driver’s intention to underestimate the risk, verifying the hypotheses H1, H2, and H3. The stronger a driver’s intention to underestimate the risk (β = 0.341, *p* < 0.001), the more likely he/she is to engage in underestimated risk behavior, verifying the hypothesis H4. Normlessness has a strong positive effect on driver’s attitude to underestimate the risk (β = 0.619, *p* < 0.001) and exhibit underestimated risk behavior (β = 0.577, *p* < 0.001), verifying H5 and H6. In addition, Figure 3 shows that the irregularity has the greatest direct predictive effect on the behavior; perceptual behavioral control has the greatest direct predictive effect on intention. This shows the applicability of the TPB theory on the one hand, and the strong support for the relationship between the added variable (normlessness) and behavioral attitudes and behavior in the context of driver underestimation of risky behavior on the other hand.

### 4.6. Effect of Demographic Variables

Table 8 presents the results of the one-way ANOVA. From the data listed in Table 8, at a confidence interval level of 95%, there was no significant difference between the gender, age, and education level in a driver’s underestimation of risk behavior (Sig. > 0.05), whereas there was a significant difference in a driver’s underestimation of the risk behavior (Sig. ≤ 0.05) in terms of the miles driven, insurance status, and accident experience.

A homogeneity of the variance test was performed on the driver’s annual mileage, and the significant value was 0.351, indicating homogeneity in the variance. The least significant difference (LSD) method was used to compare the means of each group. The results showed that drivers with a higher annual mileage had lower means in underestimating the risk behavior than drivers with a higher annual mileage, indicating that drivers with a lower annual mileage were more likely to underestimate the risk behavior. This indicated that drivers with a low annual mileage tended to underestimate the risk. The results showed that drivers with insurance other than mandatory insurance had higher mean values for underestimating the risk, indicating that drivers with a full range of insurance tended to underestimate the risk. The results of the independent sample t-test on whether the driver had accident experience showed that drivers without accident experience had higher means in underestimating the risk behavior, indicating that drivers without accident experience tend to underestimate the risk, whereas drivers with accident experience are more cautious and less likely to underestimate the risk.

## 5. Discussion

This study examined the phenomenon of underestimation of the driving risk, which is common among Chinese drivers. The results of the questionnaire revealed that 74.4% of drivers underestimate the driving risk. The ETPB model helped understand the factors influencing risk underestimation and the extent of their influence. The results of the ETPB model showed that psychological factors and personality traits significantly influence risk underestimation, in addition to driving experience, accident experience, and insurance status. Therefore, driver underestimation risk interventions should be developed from multiple perspectives to reduce the risky driving behaviors, thereby decreasing traffic accident rates and improving road safety.

The results of this study further demonstrated the applicability of the TPB in explaining driving behavior. The ATT, SN, and PBC had a significant positive effect on the driver’s intention to underestimate the risk, with standardized path coefficients of 0.32, 0.21, and 0.39, respectively. The results indicated that the PBC had the greatest effect on the intention to underestimate the risk, followed by the ATT, suggesting that drivers who are overconfident in their driving skills and do not pay attention to traffic safety will exhibit more frequent aggressive driving behaviors, easily causing traffic accidents. Moreover, if a driver believes that he/she is a skilled driver who can handle dangerous situations, then he/she will underestimate the risk [26]. On the contrary, if a driver can objectively evaluate his/her driving ability and has a late negative attitude toward underestimating the risk, then he/she will not exhibit underestimated risk behaviors with greater risk. The relationship between SN and drivers’ intention to underestimate the risk was relatively weak compared with the relationship between ATT and intention to underestimate the risk and PBC and intention to underestimate the risk, which is similar to the findings of Qi et al. [23] regarding driver’s lane grabbing behavior; these authors found that drivers’ intention to lane grab was less influenced by social norms than by attitudes and perceptual behavioral control, suggesting that drivers do not value their friends’ and family’s opinions on whether to underestimate the risk. Armitage and Commer found that the weakest predictive effect of the SN on behavioral intentions may be due to the poor measurement methods and the fact that the conceptual definition of the SN does not effectively reflect the social influence on individual behavior [25]. In the TPB, the SN reflects social pressure, which is difficult to obtain directly from whether or not to comply with the wishes of others. In addition, the underestimation of risk intention has a significant effect on drivers’ underestimation of the risk, i.e., the stronger the driver’s intention to underestimate the risk, the more likely the driver to exhibit such behavior. These results suggest that interventions for drivers’ intention to underestimate the risk should be conducted from multiple perspectives, with driver attitude and perceptual behavioral control as the main parameters, and complementary measures from subjective norms.

The introduction of N was mainly to consider personality differences in a driver’s normlessness, and the results showed a significant positive effect of N on driver’s ATT for underestimating the risk as well as URB, a conclusion consistent with the findings of Ulleberg, Nordfjærn and Şimşekoğlu. These studies showed that people who scored higher in normlessness perceived a lower risk of traffic accidents and who had a traffic safety showed negative attitudes and frequently engaged in risky driving behaviors [40,41]. Compared to the ATT, SN, PBC, and IN, N had a greater degree of influence on URB, which shows that driver normlessness is a factor that cannot be ignored in the analysis of a driver’s underestimation of risky behaviors. Drivers who scored high in normlessness were found to frequently violate traffic rules and were more likely to underestimate the driving risk because such drivers do not care much about traffic rules and are more likely to engage in aggressive driving behaviors [40]. N had a positive influence on URB and could also influence a driver’s underestimation of the risk by positively influencing the underestimating risk attitude, playing an indirect role in underestimating the risk behavior. This may be due to the fact that drivers with higher normlessness scores have more negative traffic safety attitudes and more pronounced intentions to violate the rules and are more likely to exhibit risky behaviors.

The results of this study also show no significant effect of gender or age on a driver’s tendency to underestimate the risk. This is in contrast to the findings of Rhodes, Ambros, and Griffin et al., who found that young male drivers typically underestimate potential risks in a traffic environment and are more likely to engage in unsafe driving behaviors [5,6,7]. The reason for this bias may be that their studies considering professional drivers were limited to mainly male populations and that women drive much less than men due to cultural and other factors. However, Cox found no significant correlation between driver age and risk assessment [13]. In addition, individual driver characteristics, such as the annual mileage, insurance purchase status, and accident experience, had significant effects on a driver’s tendency to underestimate the risk. In particular, drivers with small annual mileage and limited experience tend to underestimate the risk in traffic situations and have a weaker risk perception. This is consistent with the findings of Machin, who found that drivers with limited experience underestimate the risks involved in driving [55]. As a result, novice drivers with limited experience have higher accident rates and risk-taking tendencies [56]. Drivers who are well insured do not have to worry about compensation after an accident and are more likely to underestimate the risk. Drivers who have experienced accidents are more alert to dangerous scenarios while driving and are always on the lookout for hazards; in contrast, drivers without such experience are less likely to identify potential risks in traffic scenarios and are more likely to underestimate driving risks. These results suggest that the focus should be on the group of drivers with less driving experience, full insurance coverage, and no accident experience. Effective recommendations should be made for these driver groups to reduce the intention and behavior of underestimating the risks and improve road safety.

The model results show that psychological factors and personality traits have significant effects on underestimated risk behavior. Therefore, driver underestimation risk interventions should be developed from a comprehensive multifaceted perspective to reduce such behaviors and thus decrease traffic accident rates.

Driver risk perception ability is closely related to traffic accidents [3,57], and compared to experienced drivers, novice drivers have a poor risk perception ability and are unable to detect potential hazards in a road environment in a timely manner [3]. Evidently, to fundamentally reduce the occurrence of traffic accidents, it is necessary to find ways to improve a driver’s risk perception. Spolanderl referred to a driver’s ability to perceive traffic risks and prevent accidents in advance as defensive driving skills, which are mainly enhanced by actual road driving experience [58]. Foreign studies have demonstrated that incorporating defensive driving skills into novice driver training can help reduce traffic accidents by approximately 11.3% [3,59]. Currently, driver training in China focuses on theoretical regulations and basic vehicle operation skills, but ignores the learning of risk perception skills. In the future, risk perception tests should be incorporated into driver training programs to improve the risk perception skills of novice drivers.

Perceptual behavioral control has the greatest effect on a driver’s intention to underestimate the risk, suggesting that a driver’s overconfidence in their own driving skills can cause them to underestimate potential risks in a traffic environment and increase their intention to drive dangerously. Conventional driver training, which focuses on improving a driver’s technical driving skills, does not allow drivers to properly assess their own driving skills. Evans found that improvements in technical driving skills may lead to an increase in driver risk-taking behavior [26]. Therefore, driver training should not only focus on technical driving skills training, but should also help increase training in cognitive deficits so as to provide drivers with technical driving skills training without increasing overconfidence [23]. Specifically, commentary driving (CD) can be incorporated into the driver training process, where drivers explain possible risks and countermeasures that should be taken to the instructor while driving; this approach would allow drivers to properly assess their driving skills and encourage them to drive with a greater margin of safety [26,60].

Normlessness has the greatest impact on a driver’s underestimation of risky behaviors; hence, the focus should be on drivers with high normlessness scores to change their attitudes toward traffic safety. Drivers who underestimate the risks are more likely to engage in aggressive driving behaviors that can lead to traffic accidents; however, underestimating the risks has the benefits of making the drive easier and saving travel time. Therefore, traffic management should identify drivers with high scores of normlessness, improve awareness of law compliance and safety, and develop negative attitudes toward underestimating the risks. Traffic management can measure the degree of a driver’s normlessness using the normlessness and behavior scale developed by Qu et al. [61]. In addition, drivers with complete insurance coverage and no accident experience are more inclined to underestimate the risk, and traffic management should also focus on this group to prevent them from underestimating risky behavior. Traffic management can use non-punitive strategies (e.g., education or persuasive conversation) to avoid underestimated risky behavior [23], specifically by promoting educational campaigns on the hazards of underestimated risky driving behaviors, implementing lifelong driver education, and increasing traffic safety advertisements to reduce a driver’s intention to underestimate the risk behavior [62].

This study determined the factors influencing a driver’s underestimated risk behavior and clarified the degree of influence of each factor on this behavior based on the ETPB. However, the following limitations remain. First, this study mainly used the questionnaire survey method, which is susceptible to social desirability effects and recall bias and cannot confirm the authenticity of the respondents when they filled out the questionnaire [22].Subsequently, the questionnaire can be combined with field survey or combined with driving simulation experiments, and can also collect information about drivers’ driving profiles with the use of a vehicle telematics system to analyze the relationship between driver underestimation risk behavior and psychological factors more precisely. Second, the questionnaire was designed considering the acceptability of the respondents’ time in filling out the questionnaire, and 3–4 questions were used to measure each construct. Additional questions could be added to improve the accuracy of the survey. Third, Bergomi et al. pointed out that personality traits were associated with determining stress perception; “neurotic” and “impulsive” traits were especially associated with higher stress perception, which can affect the behaviors while driving [38]. Future research could consider using stress perception as a mediating variable to study the effect of personality traits on driver risk perception. Finally, risk perception can vary according to different cultural backgrounds, highly influenced by ethnicity [45,63]. The focus of this study was to explore the underestimated risk perception characteristics of Chinese drivers and it did not consider the effects of different ethnic and cultural backgrounds on drivers’ risk perception. Future research could consider the effects of different ethnic and cultural backgrounds on driver risk perceptions, and risk perception should be improved with an adequate consideration of ethnicity and cultural background when designing specific preventive interventions to increase driver occupational health and safety. In addition, this study only used data from the non-professional drivers, which may not be applicable to the risk perception characteristics of professional drivers, therefore, conducting the posted questionnaire among professional drivers may be considered as a new issue for future studies.

## 6. Conclusions

This study extended the conventional TPB by introducing drivers’ normlessness and, combined with the SEM, verified the validity of the TPB in predicting and explaining the underestimated risk behavior. Moreover, the mechanisms underlying the effects of normlessness, attitudes toward underestimated risk, subjective norms, and perceptual behavioral control on drivers’ intention to underestimate risk were comprehensively analyzed. The results showed that underestimation attitudes and perceived behavioral control positively influenced a driver’s intention to underestimate the risk. Normlessness has a direct effect on underestimated risk behavior and can also play an indirect role in underestimating the risk behavior by positively influencing driver underestimation risk attitudes. In addition, a driver’s annual mileage, insurance status, and accident experience had significant effects on the driver’s intention and behavior of underestimating the risk. This study not only fills a gap in research related to driver risk perception, but also suggests effective interventions to reduce underestimated risk behaviors. 

## Figures and Tables

**Table 1:** Scale content and corresponding references.

Construct	Item	Content	References
Normlessness (N)	N1	Driving without getting into trouble, any driving operation is allowed.	[39,40]
	N2	As long as you are not caught by traffic police or electronic cameras, you will violate traffic laws.	
	N3	Sometimes you break the law to get to your destination faster (e.g., speeding, turning without yielding to pedestrians).	
	N4	Certain driving behaviors are incorrect even though they are not illegal or unlawful.	
Attitude (ATT)	ATT1	You think it is safe to properly assess driving risks.	[43,45]
	ATT2	You think that underestimating driving risks can lead to traffic accidents.	
	ATT3	It is more comfortable for you to underestimate the risks when driving.	
	ATT4	It is more pleasant for you to underestimate the risks when driving.	
Subjective norm (SN)	SN1	Your family members will agree that you should underestimate the risks when driving.	[43,45]
	SN2	Your family members often underestimate risks when driving.	
	SN3	Your colleagues and friends would agree that you underestimate the risk when driving.	
	SN4	Other drivers on the road underestimate the risk when driving.	
Perceived behavioral control (PBC)	PBC1	You are so confident in your driving skills that you can underestimate the risks when driving.	[43,45]
	PBC2	Whether you underestimate the risks when driving is entirely up to you.	
	PBC3	You always underestimate driving the risks unconsciously.	
	PBC4	You think it is difficult to correctly assess risks when driving.	
Intention (IN)	IN1	You underestimate the risks when you have an emergency.	[43]
	IN2	You underestimate the risk when traffic conditions are good.	
	IN3	When you are in a good mood, you underestimate the driving risk.	
Underestimating driving risks behavior (URB)	URB1	You always overtake the car in front of you even when it maintains a proper speed.	In this article
	URB2	You always fail to give way in order to make time (e.g., turning to allow a car going straight, crossing a crosswalk to allow a pedestrian to pass).	
	URB3	You are always distracted by what is going on around you while driving.	
	URB4	You drive so close to the car in front that you cannot stop when it brakes.	

**Table 2:** Reliability and validity tests of the scale.

Construct		Item	Corrected Item-Total Correlation	Cronbach’s α after Deletion of Items	Cronbach’s α	KMO	Sig.
N		N1	0.783	0.85	0.890	0.954	0.00
		N2	0.802	0.843			
		N3	0.82	0.835			
		N4	0.636	0.902			
ATT	instrumental attitudes	ATT1	0.781	0.84	0.885		
		ATT2	0.724	0.862			
	affective attitudes	ATT3	0.746	0.854			
		ATT4	0.747	0.853			
SN	injunctive norm	SN1	0.702	0.829	0.863		
		SN3	0.744	0.811			
	descriptive norm	SN2	0.689	0.834			
		SN4	0.708	0.826			
PBC	control	PBC1	0.747	0.829	0.873		
		PBC2	0.697	0.849			
	self-efficacy	PBC3	0.747	0.828			
		PBC4	0.717	0.84			
IN		IN1	0.749	0.749	0.847		
		IN2	0.703	0.703			
		IN3	0.695	0.695			
URB		URB1	0.677	0.865	0.878		
		URB2	0.761	0.833			
		URB3	0.753	0.837			
		URB4	0.758	0.834			

Note: N = normlessness; ATT = underestimate risk attitude; SN = subjective norm; PBC = perceptual behavior control; IN = underestimate risk intention; URB = underestimate risk behavior; KMO = Kaiser–Meyer–Olkin.

**Table 3:** Extended TPB model bivariate correlation results.

Construct	1	2	3	4	5	6
1 N	1					
2 ATT	0.508 **	1				
3 SN	0.611 **	0.610 **	1			
4 PBC	0.568 **	0.589 **	0.600 **	1		
5 IN	0.542 **	0.582 **	0.567 **	0.604 **	1	
6 URB	0.701 **	0.310 **	0.598 **	0.624 **	0.619 **	1

Note: N = normlessness; ATT = underestimate risk attitude; SN = subjective norm; PBC = perceptual behavior control; IN = underestimate risk intention; URB = underestimate risk behavior, ** Correlation significant at 1% level.

**Table 4:** Results of convergent validity.

Construct	Item	Standardized Factor Loading	CR	AVE
N	N1	0.848	0.903	0.756
	N2	0.858		
	N3	0.901		
ATT	ATT1	0.851	0.885	0.659
	ATT2	0.782		
	ATT3	0.806		
	ATT4	0.807		
SN	SN1	0.769	0.863	0.612
	SN2	0.753		
	SN3	0.826		
	SN4	0.78		
PBC	PBC1	0.815	0.873	0.633
	PBC2	0.759		
	PBC3	0.82		
	PBC4	0.787		
IN	IN1	0.863	0.848	0.651
	IN2	0.783		
	IN3	0.771		
URB	URB1	0.729	0.879	0.645
	URB2	0.827		
	URB3	0.824		
	URB4	0.827		

Note: N = normlessness; ATT = underestimate risk attitude; SN = subjective norm; PBC = perceptual behavior control; IN = underestimate risk intention; URB = underestimate risk behavior; CR= composite reliability; AVE = average variance extracted.

**Table 5:** Results of discriminant validity.

Construct	AVE	N	ATT	SN	PBC	IN	URN
N	0.756	0.869					
ATT	0.659	0.592	0.812				
SN	0.612	0.69	0.718	0.782			
PBC	0.633	0.634	0.669	0.694	0.796		
IN	0.651	0.623	0.706	0.664	0.697	0.803	
URB	0.645	0.788	0.386	0.686	0.713	0.723	0.807

Note: N = normlessness; ATT = underestimate risk attitude; SN = subjective norm; PBC = perceptual behavior control; IN = underestimate risk intention; URB = underestimate risk behavior; AVE= average variance extracted.

**Table 6:** Assessment of model suitability.

Model Fit Index	SRMR	CFI	TLI	RMSEA	NFI	GFI	$\chi^{2}/df$
Evaluation Criteria	<0.08	>0.90	>0.90	<0.08	>0.90	>0.90	<3.0
Model Index	0.077	0.945	0.937	0.064	0.912	0.902	2.489

Note: SRMR = standardized root mean square residual; CFI = comparative fit index; TLI = Tucker–Lewis index; RMSEA = root-mean-squared error of the approximation; NFI = normed fit index; GFI = goodness of fit Index; $\chi^{2}/df$ = chi-squared freedom ratio.

**Table 7:** Results of hypothesis testing.

Hypotheses	Standardized Path Coefficient	*p*-Value	Result
H1: Attitude to underestimate the driving risk positively influences intention to underestimate the driving risk.	0.323	<0.001	Supported
H2: Subjective norm positively influences the intention to underestimate the risk.	0.214	0.006	Supported
H3: Perceived behavioral control positively influences the intention to underestimate the risk.	0.387	<0.001	Supported
H4: The intention to underestimate risk positively influences a driver’s underestimation of the risk behavior.	0.341	<0.001	Supported
H5: Normlessness positively influences the attitude of underestimating the risk.	0.619	<0.001	Supported
H6: Normlessness positively influences a driver’s behavior of underestimating the risk.	0.577	<0.001	Supported

**Table 8:** Results of one-way ANOVA.

Construct	Variables	Comparison of Differences in Means	
		F	Sig.
URB	Gender	0.161	0.522
	Age group	1.947	0.102
	Annual mileage	5.108	0.001 *
	Education level	1.948	0.122
	Insurance other than mandatory insurance	2.412	0.001 *
	Accident experience	8.79	0 *

Note: URB = underestimate risk behavior; F = statistics for the F-test; Sig. = significance of difference; * Correlation significant at 5% level.

## Data Availability

The data are available upon request.

## Appendix A

**Table A1:** Demographics of the participants.

Variables	Description	Number of Participants	Percentage (%)
Gender	Male	233	63.50
	Female	134	36.50
Age group	18–30	121	33.00
	31–40	78	21.30
	41–50	74	20.20
	51–60	23	6.30
	≥61	71	19.30
Miles driven per year (km)	<10,000	37	10.10
	10,000–20,000	58	15.80
	20,000–40,000	115	31.30
	40,000–60,000	73	19.90
	≥60,000	84	22.90
Education level	Lower secondary or below	38	10.40
	Secondary education	145	39.50
	Tertiary education	184	50.10
Any insurance other than the mandatory car insurance	Yes	229	62.40
	No	138	37.60
Accident experience	Yes	148	40.30
	No	219	59.70
Frequency of risk underestimation	Never	94	25.60
	Rarely underestimate the risk	133	36.20
	Sometimes/occasionally underestimate the risk	59	16.10
	Often underestimate the risk	62	16.90
	Always underestimate the risk	19	5.20
